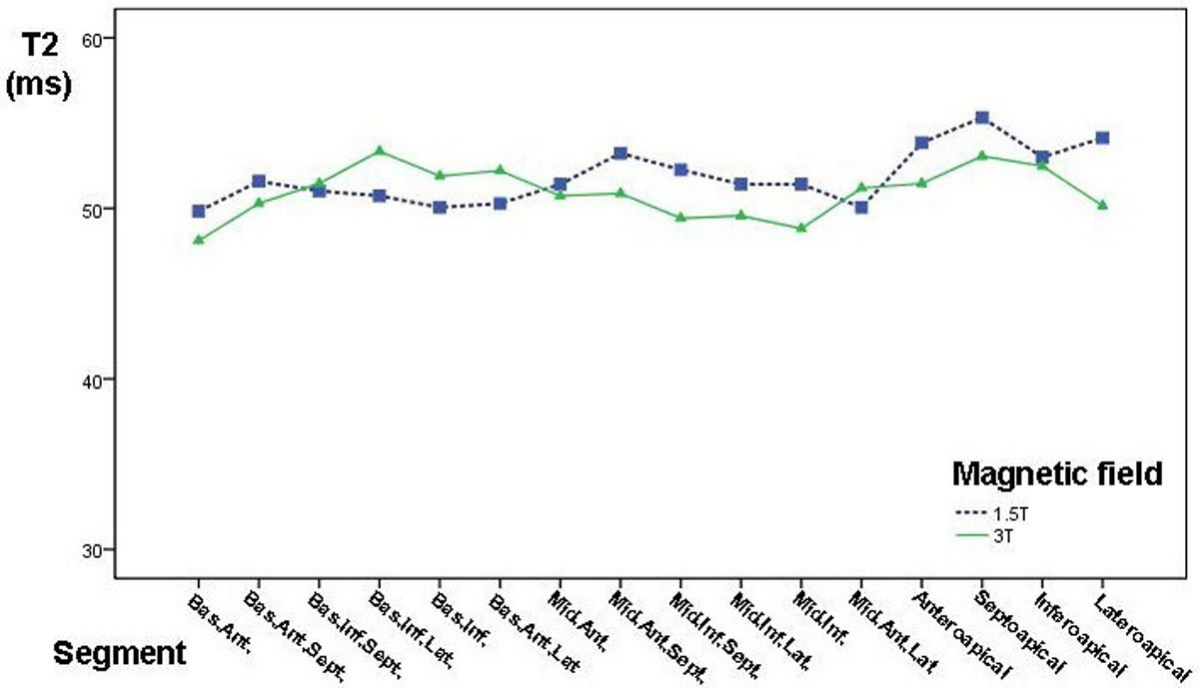# Reference values for regional and global myocardial T2 mapping with cardiovascular magnetic resonance at 1.5T and 3T

**DOI:** 10.1186/1532-429X-17-S1-P12

**Published:** 2015-02-03

**Authors:** Alicia M Maceira, Jose Vicente Monmeneu, Begoña Igual-Muñoz, Pilar M Lopez-Lereu, Pilar M Garcia, Juan Cosin

**Affiliations:** 1 Cardiac Imaging Unit, ERESA Medical Center, Valencia, Spain; 2 Cardiology, Hospital Arnau de Vilanova, Valencia, Spain

## Background

Myocardial T2 mapping has been recently developed that allows for direct measurement of local myocardial T2, overcoming some of the limitations of T2-weighted images. We aimed to obtain myocardial regional and global T2 values as a reference for normality, using a novel mapping technique both at 1.5T and at 3T.

## Methods

Between April and July 2014 we recruited 32 healthy subjects (no known cardiovascular risk factors, no previous history of heart disease), aged between 20 and 80yrs. All of them underwent a cardiovascular magnetic resonance (CMR at 1.5T, n=19; CMR at 3T, n= 13) protocol that included steady state free precession (SSFP) cines for measuring ventricular dimensions and function, TFisp 2D T2 mapping, dipyridamole stress myocardial perfusion study and late gadolinium enhancement study. T2 maps were obtained in three short axis orientations (basal, midventricular and apical). Regional myocardial T2 values were measured in a 16 segment model and global T2 values were also obtained per slice. A multifactorial ANOVA design was used for the statistical analysis.

## Results

Complete data were available for all 32 subjects (20 males, 57±12yrs). All of them had a normal CMR study, with preserved ventricular dimensions and systolic function, no regional wall motion abnormalities, no stress perfusion defects and no late gadolinium enhancement. No differences were found in regional or global T2 with respect to magnetic field (1.5T vs 3T), neither were there differences with regard to age, gender or ventricular parameters. In general, apical segments exhibited higher T2 values, but these did not reach statistical significance. Interestingly, 5 subjects had a total of 9 segments (1.7% of total segments) with T2 ≥ 70 ms, one of them was a mid-anterolateral segment and all others were apical segments. On average, normal myocardial T2 value was 52±6ms with a reference range of 40-64ms.

## Conclusions

Myocardial T2 mapping is technically feasible and easy to obtain. Small regional differences can be seen that do not reach statistical significance. Higher values can be seen in a very limited number of segments, probably attributable to acquisition quality or partial volume effects.

## Funding

N/A.

**Figure 1:**